# Comparison of peroxyacetic acid and acidified sodium chlorite at reducing natural microbial contamination on chicken meat pieces

**DOI:** 10.1016/j.psj.2023.103009

**Published:** 2023-08-09

**Authors:** Andrea R. McWhorter, Gayani Weerasooriya, Shruti Kumar, Kapil K. Chousalkar

**Affiliations:** School of Animal and Veterinary Sciences, University of Adelaide, Roseworthy, South Australia 5371, Australia

**Keywords:** chicken meat, *Salmonella*, *Campylobacter*, peroxyacetic acid, acidified sodium chlorite

## Abstract

The spin-chill process at poultry processing plants involves the immersion of chicken carcasses in cold water (<5°C) often containing sodium hypochlorite which significantly contributes to the reduction of bacterial loads. Cutting carcasses into pieces, however, has been linked with increases in *Campylobacter* and *Salmonella* counts. Here, the efficacy of PAA and ASC on reducing bacteria on skin-on, bone-in thigh cuts was investigated. Three concentrations of ASC (60, 112, and 225 ppm) and PAA (50, 75, 100 ppm) were used. Thighs were dipped into sanitizer and tested for total viable bacterial counts, *Campylobacter* load, and prevalence of *Salmonella*. The efficacy of PAA and ASC was also compared with chlorine (8 ppm). All sanitizers exhibited a greater log reduction compared with water. PAA at both 75 and 100 ppm resulted in significantly higher log reductions compared with the water only. PAA at 100 ppm and 225 ppm ASC were the most effective at reducing *Campylobacter*. All wash treatments reduced the proportion of *Salmonella* positive samples, but the greatest reduction was observed for 225 ppm ASC. Both concentrations of ASC resulted in a greater reduction in total viable counts compared with chlorine.

## INTRODUCTION

*Campylobacter* and *Salmonella* spp. often colonize the gastrointestinal tract of poultry, which ultimately represents a contamination risk for the downstream chicken meat supply chain (Hermans et al., 2012). During transport, birds experience stress which has been linked with increases in *Campylobacter* loads in the ceca (Whyte et al., 2001) and subsequently carcass contamination during processing (Hardie et al., 2019). The chicken meat industry utilizes many procedures to mitigate foodborne pathogens in the supply chain. In 1996, the Hazard Analysis Critical Control Point (**HACCP**) was implemented in the United States and was subsequently adopted by other countries to establish guidelines for the food production industry to reduce foodborne disease (Hulebak and Schlosser, 2002).

During processing, there are multiple critical control points that utilize physical or chemical interventions to minimize bacterial load on carcasses (Cox and Pavic, 2010). Postevisceration, inside-outside washers are used to reduce fecal contamination on carcasses (Cox and Pavic, 2010). Additional steps include, spin chilling which involves the immersion of chicken carcasses in cold water (<5°C) often containing chlorine (Cox and Pavic, 2010). It is important to note that the use of food processing aids, such as chlorine, in poultry meat processing is dependent on geographical location (Kwon et al., 2023). Many countries in the European Union, for example, do not permit the use food sanitizers during processing (Commission, 2004). In Australia, the majority of processing plants primarily use chlorine as an antimicrobial treatment but at concentrations less than 10 ppm (FSANZ, 2005). Poultry meat processors in the United States also use chlorine but at higher concentrations (not to exceed 50 ppm) (US Department of Agriculture and Service, 2017). In the United States, the use of peroxyacetic acid (**PAA**) is becoming more frequently used in poultry meat processing (Finstad et al., 2012; US Department of Agriculture and Service, 2017). In 2011, PAA surpassed chlorine as the most predominantly used sanitizer in postspin-chill applications (Chen et al., 2014). Several countries permit the use of acidified sodium chloride as a food processing aid (FSANZ, 2005; FDA, 2019) but has not been widely adopted.

The process of cutting whole carcasses into small cuts has been linked with increases in both *Campylobacter* (Uyttendaele et al., 1999; Habib et al., 2019) and *Salmonella* counts (Uyttendaele et al., 1999). Spray cabinets and part dips that contain sanitizers are used in several countries to control bacterial counts postcutting (Bauermeister et al., 2008). Evaluation of sanitizer effectiveness at reducing microbial contamination on cut chicken pieces, however, is challenging due to the high degree of variation in sample type, bacterial load on equipment, and sanitization parameters. In efficacy studies, drumsticks and wings have been most commonly used (Scott et al., 2015; Zhang et al., 2018; Kataria et al., 2020; Olson et al., 2020; Gonzalez et al., 2021) and are frequently obtained at different points during processing. Additionally, many sanitizer efficacy studies have experimentally inoculated chicken meat pieces with cultured bacterial strains (Scott et al., 2015; Kataria et al., 2020; Gonzalez et al., 2021). The adherence and invasion (into skin epithelia and/or pores, follicles) of cultured bacteria, however, may be different than that for bacteria that naturally contaminate chicken meat. Furthermore, bacteria that have been exposed to sanitizers during processing may be injured and therefore, would be more likely to be killed or eliminated by a postspin-chill treatment. Bacterial species evaluated also varies, with many studies focusing only on a single species. Additionally, sanitizer concentration, mode of application, duration of exposure, and pH vary from study to study.

*Campylobacter* and *Salmonella* loads vary on naturally infected pieces of chicken (McWhorter et al., 2022) yet the impact sanitization has on cuts sourced postspin chill has had limited investigation. Bacterial load can be an important factor as the bactericidal effect of sanitizers can be affected by high counts (Muhandiramlage et al., 2020). Postpackaging, the long-term disinfection efficacy of PAA and ASC is also an important consideration but has not been well characterized. Currently, Australian poultry processing plants do not use postcutting processing aids for bacterial mitigation. The objective of the present study was to investigate the potential of 2 sanitizers, peroxyacetic acid (**PAA**) and acidified sodium hypochlorite (**ASC**) as postcutting treatments for the mitigation of both *Campylobacter* and *Salmonella* on chicken pieces.

## MATERIALS AND METHODS

### Chicken Meat

Skin-on, bone-in thighs (*n* = 80), drumsticks (*n* = 80), and Maryland (combination thigh and drumstick) (*n* = 120) cuts were obtained from 2 Australian commercial poultry processing plants and tested for consistency of total viable counts, as well as *Campylobacter* load and *Salmonella* prevalence. The surface area (cm^2^) of thigh pieces was determined as per the “Australian standard for construction of premises and hygienic production of poultry meat for human consumption” (Resource Management Council of Australia and New, 2001).

Bone-in, skin-on thighs were obtained immediately after cutting and the same processing plants indicated above and used during the sanitization experiments. Thighs did not receive any additional sanitization postcutting. Prior to cutting, whole bird carcasses had gone through the processing plant spin chiller which contained chlorine (50 ppm). Thighs were placed in sterile plastic bags and transported on ice to the laboratory where they were immediately weighed and transferred to sterile resealable plastic bags for experiments.

### Sanitizers and Wash Experiments

Two sanitizers, peroxyacetic acid (**PAA**) and acidified sodium chlorite (**ASC**) were selected for efficacy testing both singly and in comparison with chlorine. A pilot study was conducted to evaluate the effects of PAA or ASC on meat appearance. Thighs were tested over a range of PAA and ASC concentrations. Meat darkening was visually observed for thighs dipped in ASC at concentrations greater than 225 ppm (data not shown) and a blanching effect was observed at concentrations over 100 ppm PAA. For the present study, 3 concentrations of each PAA (50, 75, and 100 ppm) and ASC (60, 112, and 225 ppm) were tested. For comparison with chlorine (8 ppm), 75 and 100 ppm PAA and 112 and 225 ppm ASC were used.

Prior to wash experiments, 30 L plastic drums were filled with 20 L tap water and stored at 5°C 48 h prior to experiments to allow for residual chlorine to evaporate. Prior to experiments, tap water was tested for evidence of microbial contamination and was found to be negative for bacteria (data not shown). All sanitizers were of American Chemical Standard grade. Between experiments, plastic drums were washed with hot water containing 0.3% Pyroneg (Diversy). After washing, drums were filled with 0.3% Pyroneg in hot water and soaked for 30 min prior to rinsing. Drums were rinsed thoroughly with tap water 3 times and once with reverse osmosis water.

On the day of the experiment, PAA (50 g/kg peroxyacetic acid, 250 g/kg hydrogen peroxide, 25 g/kg acetic acid; ChemSupply, Adelaide, Australia) was added to a final concentration of either 50, 75, or 200 ppm. The pH prior to washing ranged between 4.4 to 4.6 (for 50 and 75 ppm) and 4.0 to 4.2 (for 200 ppm). ASC was prepared by first acidifying 5°C tap water to pH 2.5 with 4 M citric acid (Sigma-Aldrich, Sydney, Australia). Sodium chlorite (31%, ChemSupply) was subsequently added to achieve 60, 112, or 225 ppm. The pH of all 3 ASC preparations prior to wash experiments ranged between 2.4 and 2.7. Chlorine (in the form of sodium hypochlorite, Sigma-Aldrich) was diluted to 8 ppm.

Prior to sanitization, thighs were placed individually into sterile plastic resealable bags (ThermoFisher Scientific, Sydney, Australia) each containing 100 mL buffered peptone water (**BPW**) (Oxoid, Melbourne, Australia). Thighs were manually massaged for 2 min. BPW was collected and processed for total viable count (**TVC**), *Campylobacter* loads, and the prevalence and enumeration of *Salmonella* (described in Section “Isolation and Enumeration of Bacteria”).

Thighs were then washed individually with manual agitation for 10 s in 20 L of sanitizer or plain tap water. After washing, thighs were drained for 2 min to remove excess sanitizer, placed into new resealable bags, and washed for 2 min in 100 mL BPW. Aliquots of BPW were collected and processed as above. Eight thighs were included in each wash treatment. Wash experiments were repeated 4 times (2 times for each processing plant) for a total of 32 thighs in each treatment.

The long-term disinfection efficacy of PAA and ASC on the microbial contamination of thigh cuts was also investigated. As above, thighs were first washed in 100 mL BPW. Thighs were then either briefly dipped or immersed for 10 s into either 100 ppm PAA or 225 ASC and placed into new, sterile resealable bags, and stored at 5°C. At 0, 24-, 48-, 72-, and 96-h postsanitization, thighs were rewashed with 100 mL BPW, and aliquots were processed as described above.

### Isolation and Enumeration of Bacteria

#### Total Viable Count

The aerobic total viable bacterial count (**TVC**) was determined for each wash. Serial 10-fold dilutions of BPW were prepared and 100 µL of each dilution was spread plated onto nutrient agar (Oxoid, ThermoFisher Scientific). Plates were incubated aerobically at 32°C for 18 h. Colonies were enumerated and the TVC/thigh was determined.

#### *Campylobacter* Enumeration

*Campylobacter* spp. were also enumerated from BPW wash solutions. Four hundred microliters of BPW wash were spread plate across 5 modified charcoal cefoperazone deoxycholate agar plates (**mCCDA**, Oxoid, ThermoFisher Scientific). Plates were incubated at 42°C with 10% CO_2_ for 48 h. Characteristic *Campylobacter* colonies were enumerated, and data are presented as *Campylobacter* CFU/thigh.

#### *Salmonella* Prevalence and Enumeration

To determine the prevalence of *Salmonella* on thigh samples, 50 mL of each BPW wash was incubated at 37°C for 18 h. Hundred microliters of the BPW culture was then added to 10 mL Rappaport Vassaliadis soya peptone broth (**RVS**) (Oxoid, ThermoFisher Scientific) and incubated at 42°C for 18 h. A 10 µL loopful of the RVS broth was then streaked onto xylose lysine deoxycholate (**XLD**) agar plates (Oxoid, ThermoFisher Scientific). Plates were incubated for 18 h at 37°C. A positive sample was indicated by the characteristic H_2_S producing colonies. Positives were confirmed by subculturing suspected colonies onto Brilliance *Salmonella* agar plates (ThermoFisher Scientific).

*Salmonella* were enumerated using a previously described microdilution most probable number method (Pavic et al., 2010). Briefly, 100 µL of each fresh BPW suspension was placed into microdilution tubes (SSIbio, Lodi, CA) and serial 10-fold dilutions were prepared in triplicate. 100 μL of each dilution was added microdilution tubes containing 900 μL semisolid RVS medium with the *Salmonella* selective agent (Oxoid, ThermoFisher Scientific). Samples were incubated at 42°C for 18 h. White color development indicated presumptive positive *Salmonella* growth. A combination of positive and negative microdilution tubes gave the MPN result. MPN/gram was determined using MPN tables sourced from the FDA Laboratory Methods (Blodgett, 2010).

### Statistical Analyses

Data were analyzed using GraphPad Prism Version 9.4.1 (GraphPad Software, Inc., Boston, MA). Unless indicated, all data were not normally distributed (D'Agostino-Pearson normality test) thus nonparametric statistical tests were used. Prewash and postwash data were analyzed using a Wilcoxon matched-pairs rank test. Log reduction data were analyzed using a Kruskal-Wallis test with a Dunn's comparison of the means. *P* values <0.05 were considered statistically significant.

## RESULTS

### Microbial Variation on Chicken Cuts

**Figure 1:**
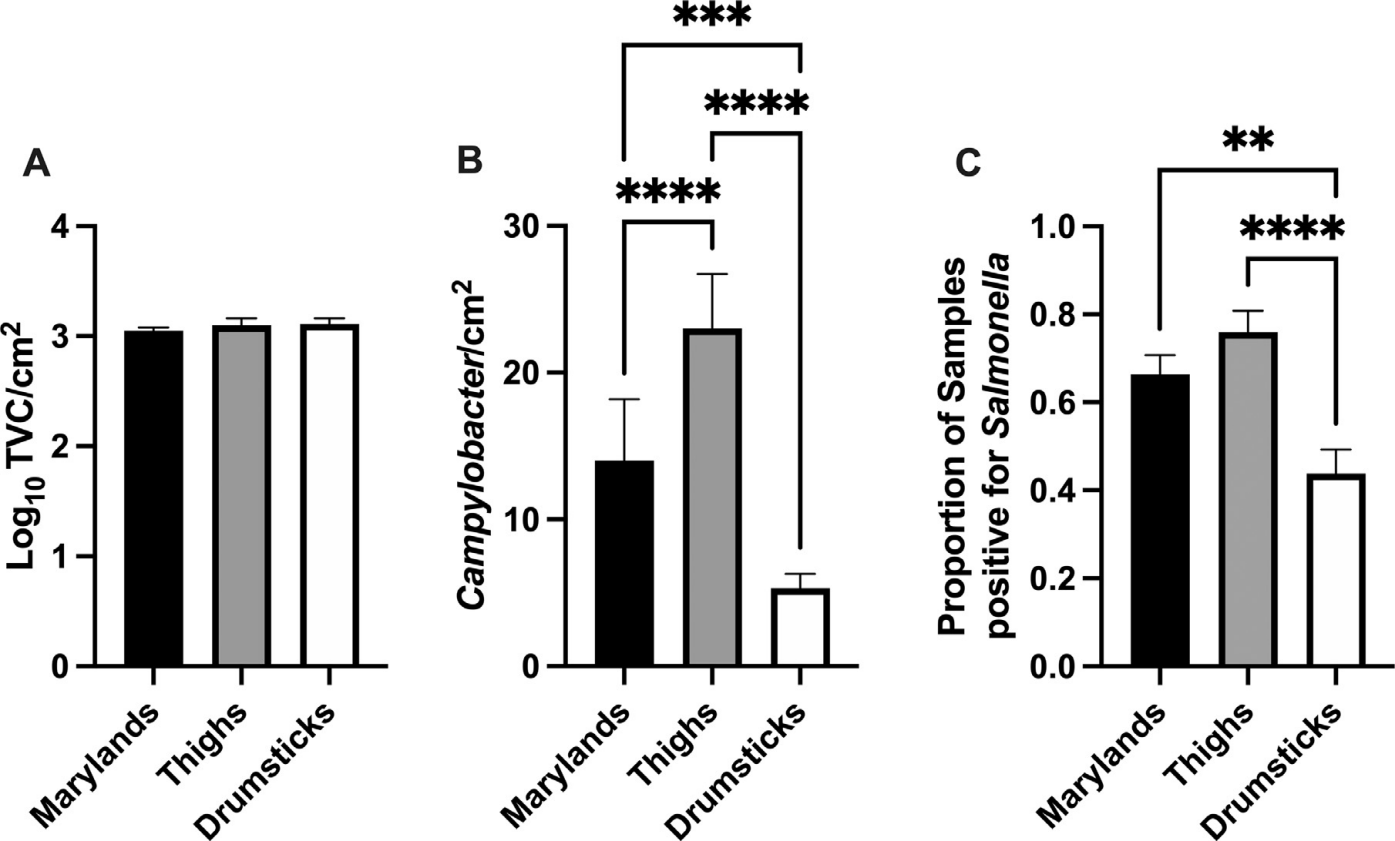
Assessment of 3 different cuts of chicken for microbial load. Maryland, thigh, and drumstick cuts were compared for TVC/cm^2^ (A), *Campylobacter*/cm^2^ (B), and the proportion of *Salmonella* positive samples (C).

TVC, *Campylobacter* load, and *Salmonella* prevalence were investigated for 3 different cuts of chicken (Maryland, thigh, and drumstick) (Figure 1A–C). No significant difference in TVC/cm^2^ was observed between cut type (Figure 1A). Thighs exhibited a significantly higher *Campylobacter* load compared with Maryland (*P* ≤ 0.0001) and drumstick (*P* ≤ 0.0001) cuts (Figure 1B). The mean *Campylobacter*/cm^2^ for thighs was 23.01 ± 3.69 while loads for the Maryland and drumsticks were 14.02 ± 4.18 and 5.32 ± 0.95, respectively. The proportion of *Salmonella* positive samples was also high for thighs (Figure 1C). The prevalence of *Salmonella* on thighs was significantly higher than drumsticks. Based on these results, bone-in, skin-on chicken thighs were used for all subsequent experiments.

### PAA and ASC Treatments Reduce TVC, *Campylobacter*, and *Salmonella* on Thighs

**Figure 2:**
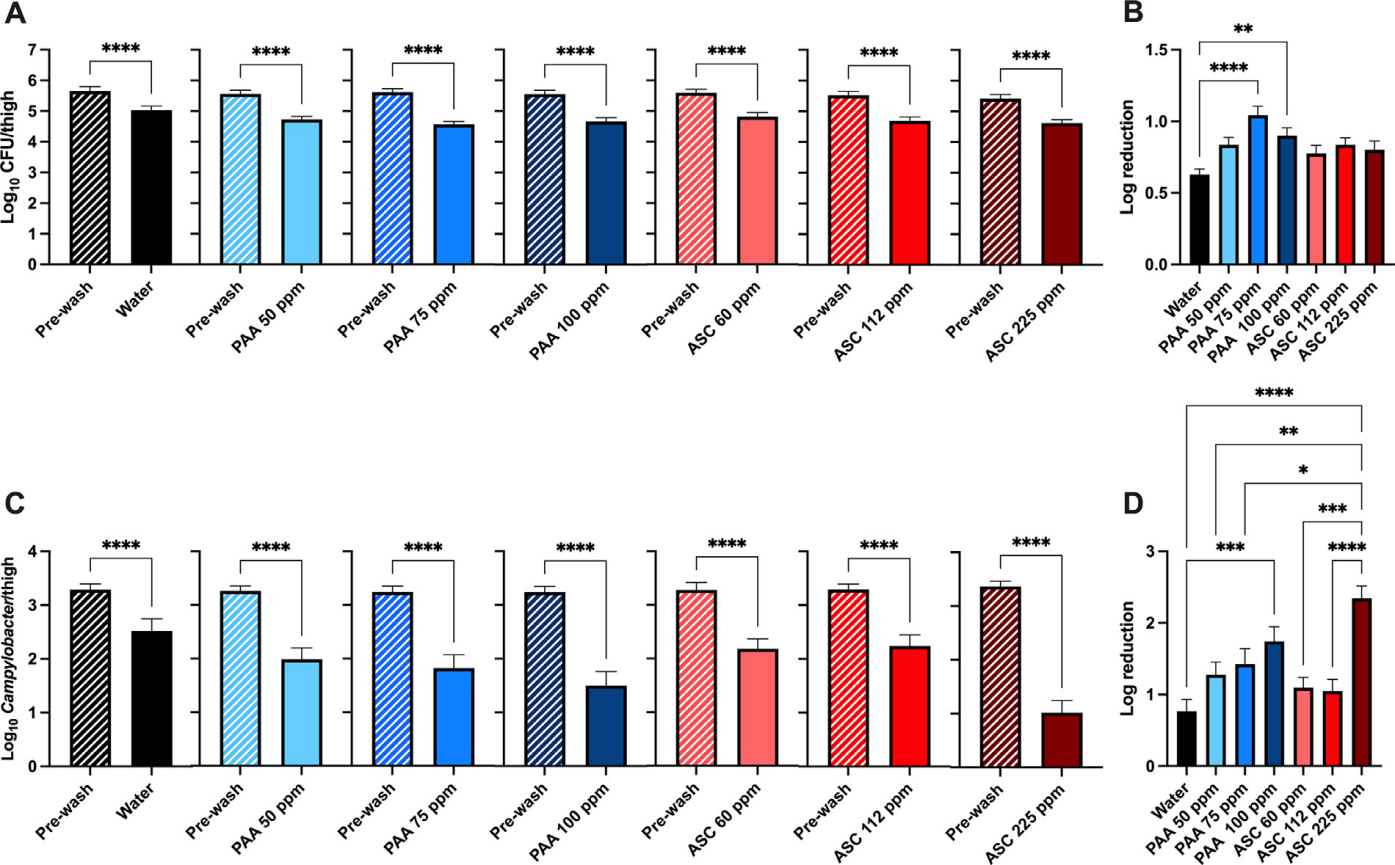
PAA and ASC reduce microbial contamination of chicken thighs. Three concentrations of PAA (50, 75, or 100 ppm) and ASC (60, 112, or 225 ppm) at 5°C were all significantly reduced the TVC (A) and *Campylobacter* (C) per thigh. Log reduction of TVC is shown in (B) and *Campylobacter* (D). The highest PAA concentrations (75 and 100) exhibited the highest log reduction in TVC/thigh and were significantly different from the water wash treatment (B). All sanitizer treatments exhibited a higher log reduction in *Campylobacter* compared with the water wash. PAA 100 ppm and ASC 225 ppm treatments had the highest overall log reduction (D).

Three concentrations of PAA and ASC were tested for their capacity to reduce the microbial load on chicken thighs. Thighs were immersed for 10 s into either tap water or sanitizers precooled to 5°C. A significant reduction in total viable bacteria was observed for all treatment groups compared with prewash counts (*P* ≤ 0.001) (Figure 2A). Log reductions are shown in Figure 2B. The 2 highest concentrations of PAA, 75 and 100 ppm, exhibited the highest log reduction of TVC. The log reduction of both 75 and 100 ppm PAA treatment groups were significantly greater than the water only treatment (*P* ≤ 0.01) (Figure 2B). No significant difference was observed between sanitizer treatments.

The effectiveness of PAA and ASC treatments to reduce *Campylobacter* on chicken thigh cuts was also determined (Figure 2C and D). Both water and sanitizer treatments resulted in a significant reduction in the mean *Campylobacter* CFU/thigh (*P* ≤ 0.01) (Figure 2C). All sanitizer treatments exhibited greater log reductions in *Campylobacter* than water (Figure 2D). The ASC 225 ppm treatment yielded the greatest log reduction of *Campylobacter* on thigh cuts (Figure 2D). The mean log reduction for ASC 225 ppm was 2.34 ± 0.17 and was significantly different from the other 2 ASC treatments, all PAA treatments, and water. PAA 100 ppm also exhibited a significantly greater reduction of *Campylobacter* compared with water (*P* ≤ 0.01) and had a mean log reduction of 1.74 ± 0.20.

**Figure 3:**
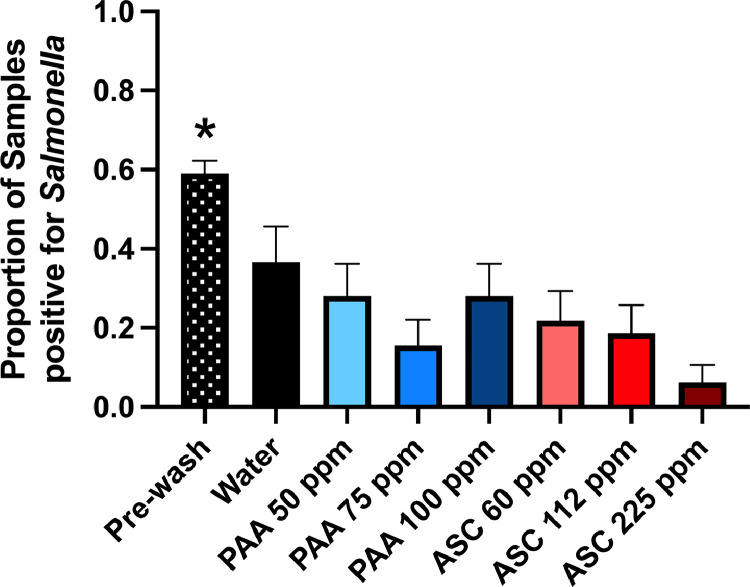
Proportion of *Salmonella* positive thighs following sanitizer dip. All dip treatments reduced *Salmonella* detected on thighs. ASC 225 ppm exhibited the greatest reduction in the proportion of *Salmonella* positive samples.

The effectiveness of PAA and ASC at reducing *Salmonella* on thighs was also characterized (Figure 3). The proportion of *Salmonella* positive samples was determined using an enrichment method and then scoring samples as either positive or negative. Both the water and sanitizer treatments resulted in a significant reduction in the mean proportion of *Salmonella* positive chicken thighs (*P* ≤ 0.01) (Figure 3). All sanitizer treatments exhibited a lower prevalence of *Salmonella* than the water only wash. The ASC 225 ppm treatment yielded the greatest reduction of *Salmonella* on thighs and was significantly different from all other sanitizer treatments (*P* ≤ 0.01). The *Salmonella* load, as determined by most probable number method, was below the limit of detection for all samples.

### Long-Term Effect of Sanitizers on Thigh Cuts Over Time

**Figure 4:**
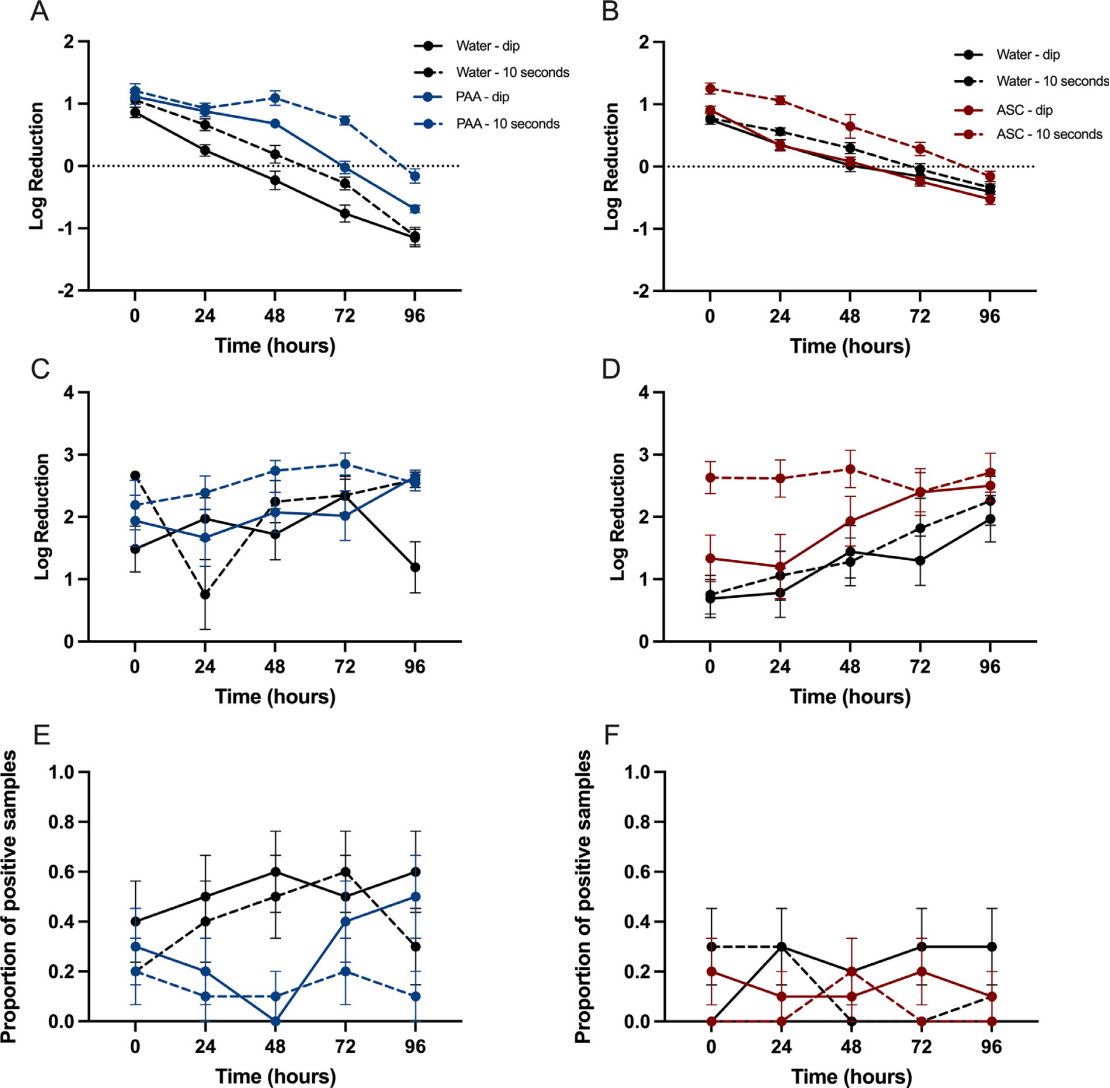
Residual effects of sanitizers. The residual effect of PAA (A, C, E) and ASC (B, D, F) on maintaining bacterial reductions on bone-in, skin-on thighs was assessed. Data are presented as log reduction ± standard error of the mean. Treatment with PAA (A) maintained a greater reduction in total viable counts compared with water. Similar results were observed for ASC (B). Log reduction of *Campylobacter* was variable post-PAA treatment (C). Treatment with ASC (D) lead to the maintenance of the greatest log reductions of *Campylobacter* over time. The proportion of samples positive for *Salmonella* was lower following both PAA (E) and ASC (F) treatments compared with water.

The long-term effects of PAA and ASC treatments on maintaining reduced bacterial loads was also investigated. Thighs were either dipped or immersed for 10 s in tap water, 100 ppm PAA, or 225 ppm ASC. Every 24 h over a 96-h period, samples were processed to determine the log reduction in TVC/thigh, *Campylobacter* CFU/thigh, or proportion of *Salmonella* positive samples (Figure 4).

For both PAA and ASC treatments, the log reduction of TVC/thigh was greatest on d 0 but declined significantly over time (PAA, *P* ≤ 0.001) (ASC, *P* ≤ 0.01) (Figure 4A and B). Water treatments in both groups did not prevent bacterial replication. The 10-s PAA and ASC immersion treatments were most effective at controlling increases in TVC on thighs over time.

The effects of residual sanitizer on maintaining reduced *Campylobacter* loads on thigh cuts was also characterized. For PAA, all treatments exhibited significant variation in *Campylobacter* load over time (*P* ≤ 0.01) but no significant difference between treatments was observed (Figure 4C). Thighs immersed for 10 s in 225 ppm ASC maintained the greatest log reduction of *Campylobacter* which was significantly different than the other treatment groups at 0-, 24- (*P* ≤ 0.001), and 48-h (*P* ≤ 0.01) post-treatment (Figure 4D). For all other treatment groups, a significant increase in log reduction was observed over time (*P* ≤ 0.01) but did not differ from each other.

The proportion of *Salmonella* positive samples was also assessed over time. Both PAA and ASC experiments exhibited variation over time in the number of samples culture positive for *Salmonella*. Both the PAA dip and 10-s immersion resulted in a lower proportion of *Salmonella* positive samples, but no significant differences were detected between the treatment groups (Figure 4E). Similar results were observed for ASC treatments (Figure 4F).

### Comparative Efficacy of Sodium Hypochlorite, ASC, and PAA on Reducing TVC, *Campylobacter*, and *Salmonella* on Thigh Cuts

**Figure 5:**
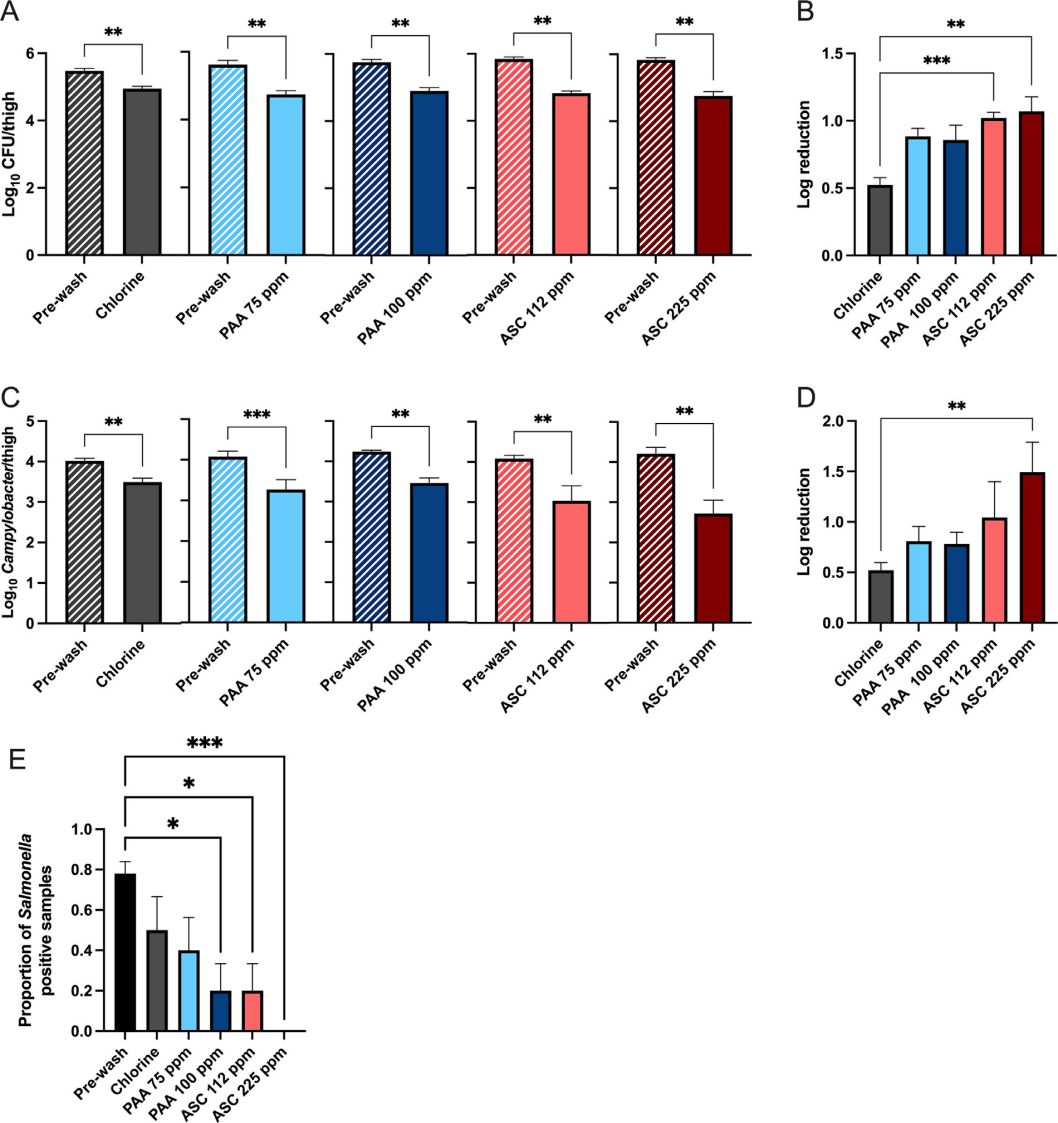
Comparison of chlorine, PAA, and ASC. Thigh cuts were washed in either chlorine (8 ppm), PAA (75 ppm or 100 ppm), and ASC (112 or 225 ppm) at 5°C. All wash treatments resulted in a significant reduction in TVC/thigh (A). ASC 112 and 225 ppm exhibited the highest log reduction in TVC/thigh and were significantly different from the chlorine wash treatment (B). A significant reduction in *Campylobacter* CFU/thigh was also observed for all wash treatments (C). ASC 225 ppm exhibited the highest log reduction in *Campylobacter* CFU/thigh and was significantly different from the chlorine wash treatment (D). Sanitizer dip treatments resulted in a reduction of *Salmonella* on thighs (E). Both ASC concentrations and PAA 100 ppm were the most effective at reducing the proportion of *Salmonella* positive thighs.

A comparison of the effectiveness of chlorine with ASC and PAA was conducted. Thighs were dipped or immersed for 10 s in either chlorine (8 ppm), ASC (112 or 225 ppm), or PAA (75 or 100 ppm). Chlorine comparison experiments were conducted at 5°C. All wash treatments significantly reduced the total viable count compared with prewash counts (*P* ≤ 0.001) (Figure 5A).

The log reduction in TVC/thigh is shown in Figure 5B. A significant effect of treatment was observed (*P* ≤ 0.001). The greatest reduction in TVC/thigh was observed for both ASC treatments with mean log reductions of 1.02 ± 0.04 for ASC 112 ppm and 1.07 ± 0.11 for ASC 225 ppm. The mean log reduction for the chlorine was 0.52 ± 0.05 and was significantly lower than both PAA 112 ppm (*P* ≤ 0.01) and ASC 225 ppm (*P* ≤ 0.001). No significant difference was detected between PAA and ASC treatment.

All treatments resulted in a significant reduction in *Campylobacter* compared with prewash counts (*P* ≤ 0.001) (Figure 5C). The log reduction in *Campylobacter* CFU/thigh is shown in Figure 5D. A significant effect of treatment was observed (*P* ≤ 0.01). The greatest reduction in *Campylobacter* loads was observed for ASC 225 ppm with a mean log reduction of 1.49 ± 0.30. The ASC 225 ppm log reduction was significantly greater than the reduction observed for the chlorine dip (*P* ≤ 0.01). No significant difference was observed between chlorine and PAA treatments.

The comparative effect of chlorine, PAA, and ASC wash treatments on the proportion of *Salmonella* positive thighs was also determined (Figure 5E). The prewash mean proportion of positive samples was 0.78 ± 0.06. Following chlorine wash the mean proportion of *Salmonella* positive samples was 0.50 ± 0.17. The proportion of *Salmonella* positive samples was 0.40 ± 0.16 for 75 ppm PAA, 0.20 ± 0.13 for 100 ppm PAA, 0.20 ± 0.13 for 112 ppm ASC, and 0.00 ± 0.00 for 225 ppm ASC. A significant effect of treatment was observed (*P* ≤ 0.01). Both concentrations of ASC and PAA 100 ppm exhibited a significantly greater reduction in the proportion of *Salmonella* positive samples compared with the prewash assessment (*P* ≤ 0.001) (Figure 5E). No significant difference was detected between sanitizers.

## DISCUSSION

The present study investigated the efficacy of different concentrations of PAA and ASC using dip or short immersion treatments on chicken cuts. A key feature of this study is that the efficacy of sanitizers was tested against naturally existing bacterial loads present on thigh cuts obtained from commercial processing plants. Bone-in, skin-on thighs were selected for use due to the consistency of *Campylobacter* and *Salmonella* loads. These results are consistent with a recent Australian study which showed the prevalence of *Campylobacter* was highest on thighs and wings (Walker et al., 2019).

PAA (McEntire et al., 2014; McWhorter et al., 2022) and ASC (Sexton et al., 2007; Chousalkar et al., 2019; McWhorter et al., 2022) have been previously shown to be effective at reducing microbial loads on chicken meat. In this study, 75 and 100 ppm of PAA resulted in the greatest reduction in bacterial counts on the surface of naturally contaminated thighs obtained postcutting. The highest concentration of PAA led to a significantly greater reduction in *Campylobacter* as well as the prevalence of *Salmonella*. Kataria et al. (2020) also demonstrated that immersion for 10 s in either 50 or 500 ppm PAA significantly reduced *Salmonella* on experimentally infected chicken wings but the effect was greater at the higher concentration. Similar results have been shown for chicken wings inoculated with multiple *Salmonella* serotypes (Scott et al., 2015). PAA has also been shown to significantly reduce artificially inoculated *Campylobacter* on chicken wings obtained postspin chill (Zhang et al., 2018; Gonzalez et al., 2021).

The highest concentration of ASC included in the present study exhibited the greatest log reduction in *Campylobacter* and *Salmonella* compared with other treatments. These results are consistent with previous ASC efficacy experiments using chicken legs obtained postevisceration (del Río et al., 2007). Interestingly, Zhang et al. (2018) showed that PAA was more effective than ASC at reducing *Campylobacter* and *Salmonella* on artificially inoculated chicken breasts and drumettes.

Postpackaging control of bacteria is also an important feature of effective microbial control. Many studies investigating the extended effects of sanitizers on chicken meat have included limited time periods (24–30 h) (Antonelli et al., 2006; Gonzalez et al., 2021). In the present study, the residual effects of PAA and ASC were assessed over 96 h. Both PAA and ASC provided more effective control of total viable counts, compared with water only treatment. Treatment with ASC was more effective than PAA at reducing total viable counts. This observation is important in relation to the shelf life of the product. Many bacterial species associated with food spoilage, however, can replicate at temperatures less than 5°C.

Treatment with PAA and ASC also initially reduced *Campylobacter* counts. The 10-s dip in ASC resulted in the greatest reduction in *Campylobacter* but stabilized. This is likely due to temperature and aerobic stress experienced by *Campylobacter*, regardless of treatment. Long-term effects of PAA and ASC on *Salmonella* loads on thigh pieces over time did not significantly vary between sanitizer and water treatments. Similar long-term bacterial reductions were observed for chicken legs artificially inoculated with multiple foodborne bacterial species and treated with PAA or ASC (del Río et al., 2007). *Salmonella* species can, however, replicate albeit slowly at low temperatures in residual chicken meat juice (Weerasooriya et al., 2022). Therefore, the long-term effects of sanitizers on controlling *Salmonella* on chicken cuts over longer periods, especially postpackaging, is warranted.

The efficacy of PAA and ASC was also compared with chlorine (sodium hypochlorite) at reducing bacterial loads on chicken pieces. All treatments reduced TVC, *Campylobacter*, and the proportion of *Salmonella* positive samples. Treatment with the highest concentration of ASC resulted in significant reductions in bacterial loads compared with chlorine. Previous investigation has shown that treatment with ASC induces significant bacterial cell damage and induces bacterial death (Weerasooriya et al., 2021). Following exposure to sodium hypochlorite, however, *Campylobacter* induces stress responses and exhibits the capacity for recovery if the favorable environment is provided (Muhandiramlage et al., 2020; Weerasooriya et al., 2021).

It should be noted that a limitation to the present study is the use of non-neutralizing buffered peptone water for rinsing. Neutralizing buffered peptone water contains soy lecithin and sodium thiosulfate which have been shown to inhibit the bactericidal effects of carry over sanitizer leading to improved detection of bacteria (Gamble et al., 2017; Vuia-Riser et al., 2018). In the present experiments, however, thigh pieces were drained for 2 min following which, thighs appeared dry. Previous studies have not drained meat samples which contributes to sanitizer carry over in the media rinse (Gamble et al., 2017). While acknowledge that the use of BPW may have contributed to a lower recovery of bacteria in these experiments, we believe this drain period minimized any residual sanitizer on thigh cuts.

The present study has shown that both chlorine and PAA reduced bacterial loads but ASC provided the greatest antimicrobial effect that persisted over time. Australian Standards for processing of meat, although outcomes based, are still prescriptive on how to achieve outcomes. The results presented here are useful for both processors and auditors to be able to vary sanitization requirements by seeking the approval of the Controlling Authority responsible for the enforcement of the Standard in each State or territory. Outcomes from this study are useful to the industry for implementation of sanitizer use on a broader scale.
